# 2-Benzyl­sulfanyl-*N*-(1,3-dimethyl­imidazolidin-2-yl­idene)aniline

**DOI:** 10.1107/S1600536813007101

**Published:** 2013-03-20

**Authors:** Ulrich Flörke, Adam Neuba, Gerald Henkel

**Affiliations:** a Universität Paderborn, Fakultät für Naturwissenschaften, Department Chemie, Warburger Strasse 100, 33098 Paderborn, Germany

## Abstract

The mol­ecular structure of the title compound, C_18_H_21_N_3_S, shows a twisted conformation with a dihedral angle of 67.45 (4)° between the aromatic ring planes and an N—C—C—S torsion angle of −5.01 (13)°. The imidazolidine ring and the aniline moiety make a dihedral angle of 56.03 (4)° and the asscociated C—N—C angle is 125.71 (10)°. The guanidine-like C=N double bond is clearly localized, with a bond length of 1.2879 (14) Å. The C—S—C angle is 102.12 (5)° and the S—C(aromatic) and S—C bond lengths are 1.7643 (11) and 1.8159 (12) Å.

## Related literature

For a related structure, see: Neuba *et al.* (2007 ▶). For the synthesis, see: Herres-Pawlis *et al.* (2005 ▶); Lindoy & Livingstone (1968 ▶).
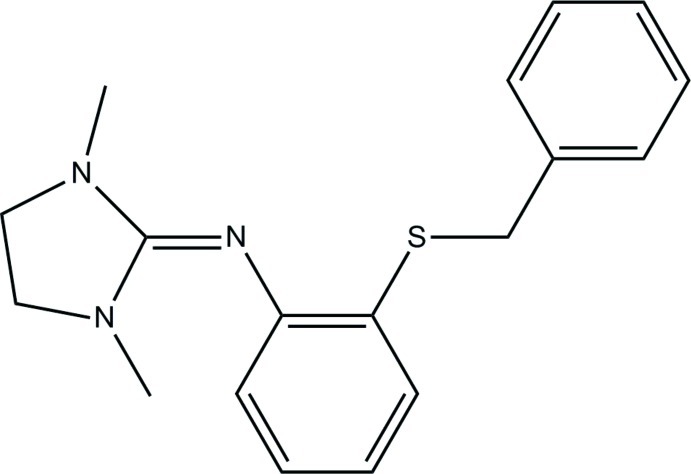


## Experimental

### 

#### Crystal data

C_18_H_21_N_3_S
*M* *_r_* = 311.44
Triclinic, 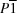

*a* = 7.9814 (5) Å
*b* = 8.1158 (5) Å
*c* = 13.9440 (9) Å
α = 97.232 (1)°
β = 102.721 (1)°
γ = 107.915 (1)°
*V* = 819.89 (9) Å^3^
*Z* = 2
Mo *K*α radiation
μ = 0.20 mm^−1^
*T* = 120 K
0.47 × 0.36 × 0.31 mm

#### Data collection

Bruker SMART APEX diffractometer
Absorption correction: multi-scan (*SADABS*; Sheldrick, 2004 ▶) *T*_min_ = 0.913, *T*_max_ = 0.941
7869 measured reflections
3959 independent reflections
3652 reflections with *I* > 2σ(*I*)
*R*_int_ = 0.016

#### Refinement

*R*[*F* ^2^ > 2σ(*F* ^2^)] = 0.035
*wR*(*F* ^2^) = 0.095
*S* = 1.04
3959 reflections
201 parameters
H-atom parameters constrained
Δρ_max_ = 0.30 e Å^−3^
Δρ_min_ = −0.21 e Å^−3^

### 

Data collection: *SMART* (Bruker, 2002 ▶); cell refinement: *SAINT* (Bruker, 2002 ▶); data reduction: *SAINT*; program(s) used to solve structure: *SHELXTL* (Sheldrick, 2008 ▶); program(s) used to refine structure: *SHELXTL*; molecular graphics: *SHELXTL*; software used to prepare material for publication: *SHELXTL* and local programs.

## Supplementary Material

Crystal structure: contains datablock(s) I, global. DOI: 10.1107/S1600536813007101/nk2203sup1.cif

Structure factors: contains datablock(s) I. DOI: 10.1107/S1600536813007101/nk2203Isup2.hkl

Supplementary material file. DOI: 10.1107/S1600536813007101/nk2203Isup3.cml

Additional supplementary materials:  crystallographic information; 3D view; checkCIF report

## Figures and Tables

**Fig. 1.**
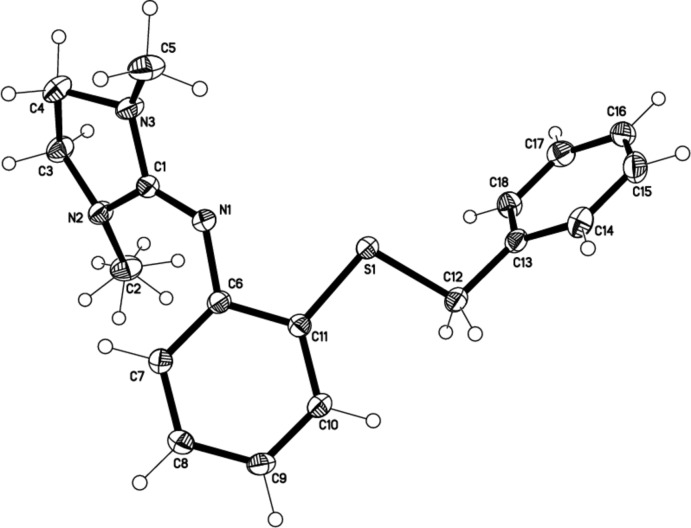
Molecular structure of the title compound with anisotropic displacement ellipsoids drawn at the 50% probability level.

## supplementary crystallographic information

### Experimental

The title compound was prepared as follows: a solution of *N*,*N*,*N*',*N*'-dimethylethylenechlorformamidinium chloride (5.07 g, 30 mmol) in dry MeCN was added dropwise to an ice-cooled solution of 2-(benzylthio)aniline (6.45 g, 30 mmol) and triethylamine (4.18 ml, 3.03 g, 30 mmol) in dry MeCN. After 3 h under reflux, a solution of NaOH (1.2 g, 30 mmol) in water was added. The solvents and NEt_3_ were then evaporated under vacuum. In order to deprotonate the mono-hydrochloride, 50 wt% KOH (aqueous, 15 ml) was added and the free base was extracted into the MeCN phase (3 *x* 80 ml). The organic phase was dried with Na_2_SO_4_. After filtration, the solvent was evaporated under reduced pressure. The title compound was obtained as white powder (yield 60%, 5.6 g). Colourless crystals suitable for X-ray diffraction were obtained by slow cooling of a hot saturated MeCN solution.

Spectroscopic data: ^1^H-NMR (500 MHz, CDCl_3_, 25°C, δ [p.p.m.]): 2.63 (s, 6H, CH_3_), 3.25 (s, 4H, CH_2_), 4.11 (s, 2H, CH_2_), 6.80 (m, 2H, CH), 7.01 (t, 1H, CH), 7.14 (d, 1H, CH), 7.21 (t, 1H, CH), 7.28 (t, 2H, CH), 7.38 (d, 2H, CH). ^13^C-NMR (125 MHz, CDCl_3_, 25°C, δ [p.p.m.]): 34.8 (CH_3_), 36.6 (CH_2_),48.5 (CH_2_) 120.5 (CH), 125.7 (CH), 126.8 (CH), 127.2 (CH), 128.3 (CH), 129.1 (CH), 129.1 (C_quat_), 137.9 (C_quat_), 148.6 (C_quat_), 155.2 (C_gua_). IR (KBr, ν [cm^-1^]): 3053 (w), 3030 (w),3003 (vw), 2933 (*m*), 2920 (*m*), 2868 (*m*), 2839 (*m*), 1954 (vw), 1973 (vw), 1635 (*versus*) (ν (C=N)), 1572 (*s*) (ν (C=N)),1493 (*m*), 1469 (*m*), 1437 (*s*), 1410 (*m*), 1394 (*m*), 1309 (w), 1281 (*m*), 1236 (*m*), 1192 (*m*), 1155 (vw), 1140 (w), 1126 (*m*),1070 (*m*), 1032 (*s*), 1003 (w), 991 (w), 970 (*m*), 920 (w), 858(w), 845(w), 816 (vw), 783 (*m*), 764 (*m*), 735 (*s*), 717 (*s*), 698 (*m*), 648 (*m*), 596 (w), 586 (w), 571 (w), 545 (w). EI—MS (m/*z* (%)): 311.2 (100) [*M*^+^], 278.2 (89), 220.2 (62) [*M*^+^—CH_2_Ph], 202.2 (43), 187.2 (96), 177.2 (40), 165.1 (83), 150.1 (29), 136.0 (52), 126.2 (47), 109.1 (33), 91.1 (55) [CH_2_Ph^+^], 70.1 (28), 56.1 (95).

### Refinement

Hydrogen atoms were clearly identified in difference syntheses, refined at idealized positions riding on the carbon atoms with isotropic displacement parameters *U*_iso_(H) = 1.2U(C_eq_) or 1.5U(–CH_3_) and C–H 0.95–0.99 Å. The hydrogen atoms of C(2) methyl group are disordered over two positions with half occupation each and refined with AFIX 123 command. All CH_3_ hydrogen atoms were allowed to rotate but not to tip.

### Crystal data

**Table:**

C_18_H_21_N_3_S	*Z* = 2
*M**_r_* = 311.44	*F*(000) = 332
Triclinic, *P*1	*D*_x_ = 1.262 Mg m^−^^3^
Hall symbol: -P 1	Mo *K*α radiation, λ = 0.71073 Å
*a* = 7.9814 (5) Å	Cell parameters from 5075 reflections
*b* = 8.1158 (5) Å	θ = 2.7–28.3°
*c* = 13.9440 (9) Å	µ = 0.20 mm^−^^1^
α = 97.232 (1)°	*T* = 120 K
β = 102.721 (1)°	Block, colourless
γ = 107.915 (1)°	0.47 × 0.36 × 0.31 mm
*V* = 819.89 (9) Å^3^	

### Data collection

**Table:**

Bruker SMART APEX diffractometer	3959 independent reflections
Radiation source: sealed tube	3652 reflections with *I* > 2σ(*I*)
Graphite monochromator	*R*_int_ = 0.016
φ and ω scans	θ_max_ = 28.1°, θ_min_ = 1.5°
Absorption correction: multi-scan (*SADABS*; Sheldrick, 2004)	*h* = −10→10
*T*_min_ = 0.913, *T*_max_ = 0.941	*k* = −10→9
7869 measured reflections	*l* = −17→18

### Refinement

**Table:**

Refinement on *F*^2^	Secondary atom site location: difference Fourier map
Least-squares matrix: full	Hydrogen site location: difference Fourier map
*R*[*F*^2^ > 2σ(*F*^2^)] = 0.035	H-atom parameters constrained
*wR*(*F*^2^) = 0.095	*w* = 1/[σ^2^(*F*_o_^2^) + (0.0478*P*)^2^ + 0.2615*P*] where *P* = (*F*_o_^2^ + 2*F*_c_^2^)/3
*S* = 1.04	(Δ/σ)_max_ < 0.001
3959 reflections	Δρ_max_ = 0.30 e Å^−^^3^
201 parameters	Δρ_min_ = −0.21 e Å^−^^3^
0 restraints	Extinction correction: *SHELXTL* (Sheldrick, 2008), Fc^*^=kFc[1+0.001xFc^2^λ^3^/sin(2θ)]^-1/4^
Primary atom site location: structure-invariant direct methods	Extinction coefficient: 0.019 (3)

### Special details

**Table:**

Geometry. All e.s.d.'s (except the e.s.d. in the dihedral angle between two l.s. planes) are estimated using the full covariance matrix. The cell e.s.d.'s are taken into account individually in the estimation of e.s.d.'s in distances, angles and torsion angles; correlations between e.s.d.'s in cell parameters are only used when they are defined by crystal symmetry. An approximate (isotropic) treatment of cell e.s.d.'s is used for estimating e.s.d.'s involving l.s. planes.
Refinement. Refinement of *F*^2^ against ALL reflections. The weighted *R*-factor *wR* and goodness of fit *S* are based on *F*^2^, conventional *R*-factors *R* are based on *F*, with *F* set to zero for negative *F*^2^. The threshold expression of *F*^2^ > σ(*F*^2^) is used only for calculating *R*-factors(gt) *etc*. and is not relevant to the choice of reflections for refinement. *R*-factors based on *F*^2^ are statistically about twice as large as those based on *F*, and *R*- factors based on ALL data will be even larger.

### Fractional atomic coordinates and isotropic or equivalent isotropic displacement parameters (Å^2^)

**Table:**

	*x*	*y*	*z*	*U*_iso_*/*U*_eq_	Occ. (<1)
S1	0.91501 (4)	0.15915 (4)	0.28380 (2)	0.02459 (10)	
N1	0.69447 (13)	0.30383 (13)	0.15949 (7)	0.0238 (2)	
N2	0.37320 (13)	0.20615 (13)	0.06598 (7)	0.0244 (2)	
N3	0.59367 (13)	0.25970 (14)	−0.01322 (7)	0.0264 (2)	
C1	0.55992 (15)	0.25990 (14)	0.07961 (8)	0.0215 (2)	
C2	0.28581 (18)	0.10816 (18)	0.13298 (10)	0.0338 (3)	
H2A	0.2394	−0.0189	0.1035	0.051*	0.50
H2B	0.1839	0.1458	0.1423	0.051*	0.50
H2C	0.3754	0.1317	0.1982	0.051*	0.50
H2D	0.2931	0.1913	0.1925	0.051*	0.50
H2E	0.3485	0.0266	0.1537	0.051*	0.50
H2F	0.1571	0.0407	0.0978	0.051*	0.50
C3	0.28106 (17)	0.14313 (18)	−0.04173 (9)	0.0303 (3)	
H3A	0.1700	0.1753	−0.0604	0.036*	
H3B	0.2470	0.0132	−0.0619	0.036*	
C4	0.42680 (17)	0.24024 (18)	−0.08879 (9)	0.0312 (3)	
H4A	0.4179	0.1694	−0.1541	0.037*	
H4B	0.4188	0.3567	−0.0983	0.037*	
C5	0.76742 (18)	0.37378 (19)	−0.02149 (10)	0.0347 (3)	
H5A	0.7645	0.4936	−0.0215	0.052*	
H5B	0.7892	0.3277	−0.0843	0.052*	
H5C	0.8661	0.3772	0.0357	0.052*	
C6	0.68392 (14)	0.34089 (15)	0.25800 (8)	0.0211 (2)	
C7	0.59065 (15)	0.44700 (16)	0.29025 (9)	0.0246 (2)	
H7A	0.5150	0.4853	0.2420	0.030*	
C8	0.60638 (16)	0.49779 (16)	0.39171 (9)	0.0258 (2)	
H8A	0.5436	0.5720	0.4124	0.031*	
C9	0.71362 (15)	0.44019 (16)	0.46273 (9)	0.0253 (2)	
H9A	0.7237	0.4742	0.5321	0.030*	
C10	0.80626 (15)	0.33282 (15)	0.43240 (8)	0.0234 (2)	
H10A	0.8782	0.2920	0.4811	0.028*	
C11	0.79447 (14)	0.28455 (14)	0.33127 (8)	0.0204 (2)	
C12	1.00985 (17)	0.08482 (16)	0.39394 (9)	0.0264 (2)	
H12A	1.0961	0.1877	0.4467	0.032*	
H12B	0.9106	0.0198	0.4214	0.032*	
C13	1.10834 (15)	−0.03469 (15)	0.36236 (8)	0.0227 (2)	
C14	1.29817 (16)	0.02421 (17)	0.38585 (10)	0.0290 (3)	
H14A	1.3675	0.1415	0.4225	0.035*	
C15	1.38726 (17)	−0.0870 (2)	0.35612 (11)	0.0347 (3)	
H15A	1.5172	−0.0460	0.3728	0.042*	
C16	1.28712 (19)	−0.25763 (19)	0.30218 (10)	0.0331 (3)	
H16A	1.3484	−0.3334	0.2816	0.040*	
C17	1.09805 (19)	−0.31805 (17)	0.27822 (10)	0.0315 (3)	
H17A	1.0293	−0.4353	0.2414	0.038*	
C18	1.00906 (16)	−0.20683 (16)	0.30818 (9)	0.0273 (2)	
H18A	0.8791	−0.2485	0.2916	0.033*	

### Atomic displacement parameters (Å^2^)

**Table:**

	*U*^11^	*U*^22^	*U*^33^	*U*^12^	*U*^13^	*U*^23^
S1	0.03082 (16)	0.02960 (16)	0.01770 (15)	0.01758 (12)	0.00528 (11)	0.00466 (11)
N1	0.0250 (4)	0.0320 (5)	0.0178 (4)	0.0139 (4)	0.0066 (4)	0.0058 (4)
N2	0.0231 (4)	0.0286 (5)	0.0197 (5)	0.0075 (4)	0.0046 (4)	0.0047 (4)
N3	0.0270 (5)	0.0335 (5)	0.0170 (5)	0.0081 (4)	0.0063 (4)	0.0050 (4)
C1	0.0250 (5)	0.0222 (5)	0.0200 (5)	0.0106 (4)	0.0072 (4)	0.0056 (4)
C2	0.0318 (6)	0.0367 (7)	0.0299 (6)	0.0042 (5)	0.0132 (5)	0.0075 (5)
C3	0.0280 (6)	0.0348 (6)	0.0222 (6)	0.0078 (5)	0.0011 (5)	0.0032 (5)
C4	0.0342 (6)	0.0365 (7)	0.0188 (5)	0.0094 (5)	0.0026 (5)	0.0067 (5)
C5	0.0334 (6)	0.0424 (7)	0.0253 (6)	0.0057 (5)	0.0134 (5)	0.0056 (5)
C6	0.0208 (5)	0.0242 (5)	0.0179 (5)	0.0076 (4)	0.0049 (4)	0.0046 (4)
C7	0.0246 (5)	0.0303 (6)	0.0225 (5)	0.0133 (4)	0.0071 (4)	0.0076 (4)
C8	0.0257 (5)	0.0295 (6)	0.0253 (6)	0.0124 (4)	0.0101 (4)	0.0043 (5)
C9	0.0260 (5)	0.0302 (6)	0.0182 (5)	0.0080 (4)	0.0073 (4)	0.0020 (4)
C10	0.0238 (5)	0.0262 (5)	0.0181 (5)	0.0080 (4)	0.0029 (4)	0.0042 (4)
C11	0.0199 (5)	0.0212 (5)	0.0196 (5)	0.0072 (4)	0.0049 (4)	0.0034 (4)
C12	0.0329 (6)	0.0291 (6)	0.0194 (5)	0.0166 (5)	0.0031 (4)	0.0051 (4)
C13	0.0264 (5)	0.0236 (5)	0.0189 (5)	0.0105 (4)	0.0041 (4)	0.0069 (4)
C14	0.0260 (6)	0.0280 (6)	0.0282 (6)	0.0061 (4)	0.0016 (5)	0.0075 (5)
C15	0.0248 (6)	0.0479 (8)	0.0356 (7)	0.0162 (5)	0.0076 (5)	0.0158 (6)
C16	0.0433 (7)	0.0430 (7)	0.0282 (6)	0.0290 (6)	0.0150 (5)	0.0153 (5)
C17	0.0413 (7)	0.0253 (6)	0.0290 (6)	0.0133 (5)	0.0096 (5)	0.0049 (5)
C18	0.0244 (5)	0.0259 (6)	0.0290 (6)	0.0071 (4)	0.0055 (4)	0.0041 (5)

### Geometric parameters (Å, º)

**Table:**

S1—C11	1.7643 (11)	C6—C7	1.3937 (15)
S1—C12	1.8159 (12)	C6—C11	1.4143 (15)
N1—C1	1.2879 (14)	C7—C8	1.3894 (16)
N1—C6	1.3953 (14)	C7—H7A	0.9500
N2—C1	1.3791 (14)	C8—C9	1.3861 (17)
N2—C2	1.4576 (15)	C8—H8A	0.9500
N2—C3	1.4652 (15)	C9—C10	1.3879 (16)
N3—C1	1.3789 (14)	C9—H9A	0.9500
N3—C5	1.4495 (15)	C10—C11	1.3896 (15)
N3—C4	1.4559 (15)	C10—H10A	0.9500
C2—H2A	0.9800	C12—C13	1.5055 (16)
C2—H2B	0.9800	C12—H12A	0.9900
C2—H2C	0.9800	C12—H12B	0.9900
C2—H2D	0.9800	C13—C14	1.3884 (16)
C2—H2E	0.9800	C13—C18	1.3928 (16)
C2—H2F	0.9800	C14—C15	1.3868 (19)
C3—C4	1.5190 (18)	C14—H14A	0.9500
C3—H3A	0.9900	C15—C16	1.383 (2)
C3—H3B	0.9900	C15—H15A	0.9500
C4—H4A	0.9900	C16—C17	1.3823 (19)
C4—H4B	0.9900	C16—H16A	0.9500
C5—H5A	0.9800	C17—C18	1.3870 (17)
C5—H5B	0.9800	C17—H17A	0.9500
C5—H5C	0.9800	C18—H18A	0.9500
			
C11—S1—C12	102.12 (5)	C7—C6—C11	118.30 (10)
C1—N1—C6	125.71 (10)	N1—C6—C11	116.83 (10)
C1—N2—C2	121.84 (10)	C8—C7—C6	121.15 (10)
C1—N2—C3	108.85 (9)	C8—C7—H7A	119.4
C2—N2—C3	116.19 (10)	C6—C7—H7A	119.4
C1—N3—C5	119.96 (10)	C9—C8—C7	120.01 (11)
C1—N3—C4	109.64 (9)	C9—C8—H8A	120.0
C5—N3—C4	118.80 (10)	C7—C8—H8A	120.0
N1—C1—N3	119.85 (10)	C8—C9—C10	119.91 (11)
N1—C1—N2	131.63 (10)	C8—C9—H9A	120.0
N3—C1—N2	108.52 (9)	C10—C9—H9A	120.0
N2—C2—H2A	109.5	C9—C10—C11	120.45 (10)
N2—C2—H2B	109.5	C9—C10—H10A	119.8
H2A—C2—H2B	109.5	C11—C10—H10A	119.8
N2—C2—H2C	109.5	C10—C11—C6	120.16 (10)
H2A—C2—H2C	109.5	C10—C11—S1	124.81 (9)
H2B—C2—H2C	109.5	C6—C11—S1	115.00 (8)
N2—C2—H2D	109.5	C13—C12—S1	107.83 (8)
N2—C2—H2E	109.5	C13—C12—H12A	110.1
H2D—C2—H2E	109.5	S1—C12—H12A	110.1
N2—C2—H2F	109.5	C13—C12—H12B	110.1
H2D—C2—H2F	109.5	S1—C12—H12B	110.1
H2E—C2—H2F	109.5	H12A—C12—H12B	108.5
N2—C3—C4	102.28 (9)	C14—C13—C18	118.95 (11)
N2—C3—H3A	111.3	C14—C13—C12	120.99 (11)
C4—C3—H3A	111.3	C18—C13—C12	120.06 (10)
N2—C3—H3B	111.3	C15—C14—C13	120.43 (11)
C4—C3—H3B	111.3	C15—C14—H14A	119.8
H3A—C3—H3B	109.2	C13—C14—H14A	119.8
N3—C4—C3	101.32 (9)	C16—C15—C14	120.07 (11)
N3—C4—H4A	111.5	C16—C15—H15A	120.0
C3—C4—H4A	111.5	C14—C15—H15A	120.0
N3—C4—H4B	111.5	C17—C16—C15	120.13 (12)
C3—C4—H4B	111.5	C17—C16—H16A	119.9
H4A—C4—H4B	109.3	C15—C16—H16A	119.9
N3—C5—H5A	109.5	C16—C17—C18	119.76 (12)
N3—C5—H5B	109.5	C16—C17—H17A	120.1
H5A—C5—H5B	109.5	C18—C17—H17A	120.1
N3—C5—H5C	109.5	C17—C18—C13	120.65 (11)
H5A—C5—H5C	109.5	C17—C18—H18A	119.7
H5B—C5—H5C	109.5	C13—C18—H18A	119.7
C7—C6—N1	124.38 (10)		
			
C6—N1—C1—N3	167.61 (10)	C8—C9—C10—C11	0.94 (17)
C6—N1—C1—N2	−13.5 (2)	C9—C10—C11—C6	−1.62 (17)
C5—N3—C1—N1	−26.12 (17)	C9—C10—C11—S1	176.39 (9)
C4—N3—C1—N1	−168.89 (11)	C7—C6—C11—C10	0.90 (16)
C5—N3—C1—N2	154.73 (11)	N1—C6—C11—C10	173.18 (10)
C4—N3—C1—N2	11.97 (13)	C7—C6—C11—S1	−177.30 (8)
C2—N2—C1—N1	−30.72 (19)	N1—C6—C11—S1	−5.01 (13)
C3—N2—C1—N1	−170.28 (12)	C12—S1—C11—C10	10.12 (11)
C2—N2—C1—N3	148.28 (11)	C12—S1—C11—C6	−171.78 (8)
C3—N2—C1—N3	8.73 (13)	C11—S1—C12—C13	176.61 (8)
C1—N2—C3—C4	−24.46 (13)	S1—C12—C13—C14	104.05 (11)
C2—N2—C3—C4	−166.57 (11)	S1—C12—C13—C18	−75.54 (12)
C1—N3—C4—C3	−26.33 (13)	C18—C13—C14—C15	−0.26 (18)
C5—N3—C4—C3	−169.58 (11)	C12—C13—C14—C15	−179.85 (11)
N2—C3—C4—N3	29.57 (12)	C13—C14—C15—C16	0.38 (19)
C1—N1—C6—C7	−45.01 (17)	C14—C15—C16—C17	−0.4 (2)
C1—N1—C6—C11	143.23 (11)	C15—C16—C17—C18	0.22 (19)
N1—C6—C7—C8	−171.15 (11)	C16—C17—C18—C13	−0.10 (19)
C11—C6—C7—C8	0.50 (16)	C14—C13—C18—C17	0.12 (18)
C6—C7—C8—C9	−1.19 (18)	C12—C13—C18—C17	179.71 (11)
C7—C8—C9—C10	0.46 (17)		
